# High-sensitivity HBV DNA test for the diagnosis of occult HBV infection: commonly used but not reliable

**DOI:** 10.3389/fcimb.2023.1186877

**Published:** 2023-05-16

**Authors:** Chengwei Wang, Rongrong Xue, Xinru Wang, Li Xiao, Jianchun Xian

**Affiliations:** ^1^ Department of Infectious Diseases, The Affiliated Taizhou People’s Hospital of Nanjing Medical University, Taizhou, Jiangsu, China; ^2^ Department of Infectious Diseases, Yancheng First People’s Hospital, Yancheng, China

**Keywords:** hepatitis B virus, high-sensitivity HBV DNA test, occult HBV infection (OBI), hepatocellular carcinoma, cirrhosis

## Abstract

Occult hepatitis B virus (HBV) infection (OBI) is a condition in which replication-competent viral DNA is detected in the liver (with detectable or undetectable HBV DNA in serum) of individual testing negative for HBV surface antigen (HBsAg). It is a risk factor for transfusion or transplant transmission, reactivation after immunosuppression or chemotherapy, and progression of chronic liver disease and hepatocarcinogenesis. The long-term stable presence of covalently closed circular DNA (cccDNA), which is fully replicative in the nucleus of infected hepatocytes is the molecular basis for the formation of OBI. HBV genome in liver tissue, HBV DNA and anti-HBc test in serum are the gold standard, common method and alternative markers for OBI diagnosis, respectively. Due to the stability of covalently closed circular DNA (cccDNA) and the long half-life of hepatocytes, the existence of OBI is extensive and prolonged. The low and/or intermittent replication of HBV in OBI patients, the limitations of the sensitivity of serological tests, and the non-standardized and invasive nature of liver histology render the “commonly used” serological tests are unreliable and the “gold standard” liver histology is impractical, thus the findings from studies on the formation, diagnosis and transplantation or transfusion transmission of HBV in OBI strongly suggest that the “alternative” marker, the anti-HBc test, may be the most reliable and practical approach for OBI diagnosis.

## 1 Introduction

Occult hepatitis B virus (HBV) infection (OBI) is generally defined as the presence of replication-competent HBV DNA (i.e., HBV covalent closed circular DNA [cccDNA]) in the liver and/or HBV DNA in the blood of individuals who test negative for hepatitis B surface antigen (HBsAg) according to currently available assays. According to the HBV-specific antibody profile, OBI can be divided into seropositive OBI with positive hepatitis B core antibody (anti-HBc) and/or hepatitis B surface antibody (anti-HBs) and seronegative OBI with negative anti-HBc and anti-HBs. Among them, seronegative OBI accounts for about 1% to 20% of all OBI cases (Raimondo et al., 2019). OBI is a risk factor for transfusion or transplant transmission, reactivation after immunosuppression or chemotherapy, chronic liver disease progression and hepatocarcinogenesis (Saitta et al., 2022). Due to the stability of covalently closed circular DNA (cccDNA) and the long half-life of hepatocytes, the existence of OBI is extensive and prolonged, while the low and/or intermittent replication of HBV in patients with OBI (Morales-Romero et al., 2014; Saitta et al., 2022), the limitations of the sensitivity of serological tests, and the non-standardized and invasive nature of liver histology determine that the “commonly used” serological tests are unreliable and the “gold standard” liver histology is impractical. The practical results of research on the formation mechanism, diagnosis and transplantation or transfusion transmission of OBI strongly suggest that although there is a certain proportion of seronegative OBI, as long as anti-HBc is positive, then there is a high possibility of the presence of OBI, and the “alternative” marker anti-HBc test is the most reliable and practical method for the diagnosis of OBI. In this article, we explore the above issues in terms of the definition of OBI, the mechanism of covalently closed circular DNA (cccDNA) clearance, the limitations of diagnostic methods and the practical findings of transplantation/transfusion HBV transmission.

## 2 Limitation of OBI serologic diagnosis method

A sufficient and necessary condition for the diagnosis of OBI is the detection of replication-competent HBV DNA in the liver and/or HBV DNA in blood of HBsAg-negative individuals. Among them, the “gold standard” for diagnosis is the detection of HBV DNA in the liver tissue, the “commonly used” method is the detection of HBV DNA in the blood, and the anti-HBc test in blood is an “alternative” method for OBI diagnosis (Raimondo et al., 2019; Saitta et al., 2022) (**Table 1**). In the serological diagnosis of OBI, many experts and guidelines currently recommend a highly sensitive HBV DNA method with a lower limit of detection (10-20 IU/ml) (which should actually be the lower limit of quantification, author’s note), a wider linear range, and a higher specificity. However, due to the fact that HBV DNA in OBI patients is usually <200 IU/ml, of which 80%-90% is <20 IU/ml, with intermittent elevation/positive characteristics, along with the impact of test efficacy and method, serum/plasma extraction volume, sample volume, genotype coverage, internal standard setting and cost differences, facing shortcomings such as nucleic acid contamination, lack of industry regulation and standardization, thus the limitations of serum HBV DNA testing for OBI diagnosis are evident (Kang et al., 2014; Morales-Romero et al., 2014; Raimondo et al., 2019; Cai et al., 2022), the most prominent of which is the sensitivity of the assay.

**Table 1:** Diagnosis of OBI.

Approaches	Notes
Detection of HBsAg in the blood	Detection sensitivity and detectivity of S-escape variants
Detection of HBV DNA in the liver tissue	Standardised and valid assays are not yet available,invasive nature
Detection of HBV DNA in the blood	Commonly used,depending on sensitivity extremely
Detection of anti-HBc in the blood	Use as a surrogate

Altunay et al. (2010) revealed that the rate of OBI detection among isolated anti-HBc positive individuals by serological methods was only 0.091% (**Table 2**). Cai et al. (2022) showed that the positive rate of HBV DNA detection in the serum of HBsAg(-)/anti-HBc(+) patients was 5.4% (108/2013)(see more details in **Table 2**). The detection rate may vary depending on the pattern of HBV serologic markers, with the highest detection rate in anti-HBe(+)/anti-HBc(+) status (15.61%, 32/205), followed by anti-HBs(+)/anti-HBe(+)/anti-HBc(+) (6.27%, 53/845), anti-HBc(+) (3.73%, 10/268), and anti-HBs(+)/anti-HBc(+) (1.87%,13/695) (**Table 3**). It is evident that anti-HBs is negatively correlated with the detection rate of OBI serologically, while anti-HBe is positively correlated with the detection rate of OBI serologically, which is also consistent with the role of anti-HBs and anti-HBe, since anti-HBs is a protective antibody, whereas anti-HBe is a sign that the body has a strong immune response and is still in the immune clearance or recovery phase (de Almeida Pondé, 2021). Candotti et al. (2019) showed that HBsAg, HBV DNA-negative donors were still at risk of transfusion-transmitted HBV even after 3 repeated tests using the most sensitive serologic method [lower limit of detection (LOD): 3.4 IU/mL], and reducing transfusion volume and receptor anti-HBs positivity contributed to prevention. The intermittent positivity of serum HBV DNA in patients with OBI shown in the evaluation of longitudinal studies also suggests that serum HBV DNA negativity does not exclude the existence of OBI even using the most sensitive serological methods. The sensitivity of quantitative serum HBV DNA testing for the diagnosis of OBI is not satisfactory, i.e., serologic methods, although commonly used, are not reliable.

**Table 2:** Summary of case reports.

First author	Year	Country or region	Design	Cumulative cases	Followup	Incidence of OBI,%
serological methods						
Altunay (Altunay et al., 2010)	2010	Turkey	Retrospective	2,748	NR	0.091%
Cai (Cai et al., 2022)	2022	China	Retrospective	2,013	NR	5.4%
liver histology VS serological methods						
Yuki, N (Yuki et al., 2003)	2003	Japan	Retrospective	13	4.2years	22.22%VS21.43%
Komori, M (Komori et al., 2001)	2001	Japan	Retrospective	15	4.4years	100%VS13.33%
Bréchot, C (Bréchot et al., 2001)	2001	–	Review	–	–	94%VS28%
Knöll A (Knöll et al., 2006)	2006	Germany	Retrospective	545	1.2years	40.54%VS8.1%
2 different liver histology approaches VS 1serological method						
Caviglia GP (Caviglia et al., 2018)	2018	Italy	Retrospective	100	NR	nested PCR 52% digital droplet PCR 52% VS real-time PCR 22.2%

NR, not reported.

**Table 3:** OBI incidence depending on HBV serology makers.

HBV serology status	Number of total patients (n)	Incidence of OBI %
HBeAb/HBcAb-positive	205	15.61%
HBsAb/HBeAb/HBcAb-positive	845	6.27%
HBcAb-positive	268	3.73%
HBsAb/HBcAb-positive	695	1.87%

HBV, hepatitis B virus; HBsAb, hepatitis B surface antibody; HBcAb, hepatitis B core antibody; HBeAb, hepatitis B e antibody.

## 3 The mechanism of cccDNA clearance determines the long-term existence of OBI

The long-term stable presence of fully replicative cccDNA in the nucleus of hepatocytes is the main molecular basis of OBI (Zoulim, 2005). It is generally accepted that there are two balances in infection of HBV in human body, one between viral replication in hepatocytes and viral decline in circulation and the other between replenishment and decline of the cccDNA pool (Zeuzem et al., 1997; Murray et al., 2006; Dandri et al., 2008). The host immune function and drug interventions can affect the first balance, but hardly the second (Zeuzem et al., 1997; Murray et al., 2006; Dandri et al., 2008). Due to different clinical settings or host immune status, the half-life of cccDNA can range from 1 month to 26.2 months (Zeuzem et al., 1997; Murray et al., 2006; Dandri et al., 2008), and the half-life of HBV-infected hepatocytes can range from 10 to 100 days (Nowak et al., 1996). The incompleteness and lack of long-term effectiveness of host immune control and current antiviral drug treatment determine that virus DNA synthesis in hepatocytes can still not be completely blocked even with highly effective antiviral drug treatment. HBV DNA can still be detected in the liver even 30 years after HBsAg clearance or seroconversion (Zeuzem et al., 1997; Bläckberg and Kidd-Ljunggren, 2000; Murray et al., 2006; Dandri et al., 2008; Bes et al., 2012; Boyd et al., 2016), so once HBV infection is established, the cccDNA in liver or OBI status can persist for a long time or even for life.

## 4 The efficiency of serological methods for the diagnosis of OBI significantly lower than that of liver histology

A study from Japan showed that in a follow-up of 14 cases of acute self-limiting HBV infection with a median time of 4.2 years (range 1.8 to 9.5 years) after recovery, the serum positivity rate was 21.43% (3/14) by real-time polymerase chain reaction (PCR) detection (detection limit of 10 copies/ml). HBV cccDNA was detected in biopsy liver tissues (100%) in all 9 patients. However, only 2 out of 9 patients with positive HBV DNA in their liver tissues had positive serum results (2/9, 22.22%) (Yuki et al., 2003) (**Table 2**). Another study from Japan showed that in a follow-up of 15 patients with chronic hepatitis B after HBsAg seroclearance with a median follow-up time of 4.4 years (range 0.9 to 15.3 years), the serum occult hepatitis B infection (OBI) detection rate was 13.33% (2/15) by real-time PCR detection (quantitative lower limit of 200 copies/ml). However, HBV DNA was detected in all liver tissues of the patients examined (100%) (Komori et al., 2001) (**Table 2**). A review from Paris, France showed that the positivity rates of viral genome in serum and liver were 28% and 94%, respectively (Bréchot et al., 2001) (**Table 2**). A study from Italy showed that among 100 HBsAg-negative and anti-HBc-positive liver transplant donors (mean age 68.2 years, 64 males, 36 females), nested PCR detection of HBV DNA in liver tissue revealed that 52% (52/100) of the individuals had occult hepatitis B infection (OBI) (defined as positive for nested PCR in at least 2 different HBV genome regions). Among the 52 individuals who tested positive for nested PCR in liver tissue, digital droplet PCR (ddPCR) was used to detect cccDNA in liver cells, and the positivity rate was 52% (27/52). Using real-time PCR, only 22.2% (6/27) of individuals who tested positive for cccDNA by ddPCR had positive HBV DNA in their blood (all <20 IU/ml) (Caviglia et al., 2018), indicating that the sensitivity of serological methods for diagnosing OBI is significantly lower than that of ddPCR and nested PCR techniques in liver tissue (**Table 2**). Knöll A et al. (Knöll et al., 2006). (**Table 2**) examined serologic HBV DNA in 545 patients with isolated anti-HBc(+) and liver tissue HBV DNA in 37 patients with negative serum HBV DNA but isolated anti-HBc(+). The results showed a serology positivity rate of 8.1% (44/545) with a detection limit of 50-100 copies/ml. The highest positivity rate was observed in the age group of 1-30 years (4/27; 14.8%), while the lowest was observed in the age group of >80 years (1/21; 4.8%), but with a non-linear relationship between age and positivity rate. Among the 37 patients with HBV DNA negative serology, 15 (40.54%) were found to be HBV DNA positive in liver tissue samples, indicating that even in cases where the serological detection rate is 0, the liver tissue positivity rate can reach 40% as long as the serum anti-HBc is positive. These findings suggest that the serology method has a lower positivity rate (8.1-28%) compared to the liver histology positivity rate (50-100%).

The above comparison study of serology and liver histology suggests that the positivity rate of serology for diagnosing occult hepatitis B infection (OBI) is only about 10%-20% of that of liver histology. Additionally, the age of the host and the mode of prior HBV infection are also related to the detection rate of OBI.

## 5 HBsAg(-)/anti-HBc(+) liver donor transmitting HBV indicates that anti-HBc(+) is the most reliable (up to 90%) serum marker for OBI

A study in Spain retrospectively reviewed 25 cases of liver transplant recipients with negative HBV markers between 1995 and 1998 who received liver from donors positive for HBsAg (-)/anti-HBc (+). The results showed that 15 cases (60%, 15/25) developed hepatitis B (confirmed by detection of HBsAg in two consecutive serum samples) (Prieto et al., 2001) (**Table 4**). In the study by Uemoto et al (Uemoto et al., 1998), 16 cases of anti-HBc (+)/anti-HBs (+) (14 cases also positive for anti-HBe) donor livers were transplanted to recipients who were negative for anti-HBs and lacking prophylaxis. Among the recipients, 15 cases (93.75%) showed positive HBsAg in their serum (**Table 4**). In 50%-73% of anti-HBc (+) liver donors, HBV DNA was detected in liver, but all donors had negative HBV DNA in their serum. Anti-HBc (+) is a risk marker for HBV transmission and reactivation after liver transplantation (Uemoto et al., 1998). The risk of HBV transmission from the anti-HBc(+) liver donors to the recipients ranged from 25% - 95%, and the presence of anti-HBs from the donors did not appear to provide good protection against HBV transmission to the recipients (Uemoto et al., 1998; Prieto et al., 2001; Muñoz, 2002). The information provided by these studies strongly suggests that the absence of serum HBV DNA in anti-HBc (+) donors does not rule out the possibility of HBV transmission to the recipient. In addition, the synthesis of cccDNA and HBcAg is closely related to the production of anti-HBc (Moretto et al., 2020). Therefore, the detection of anti-HBc in blood can be used as an alternative biomarker for OBI, and as a more reliable marker for the diagnosis of OBI. This may also be a key reason why the 2017 EASL (European Association for the Study of the Liver et al., 2017) and Pollicino et al (Pollicino and Caminiti, 2021). consider HBsAg-negative/anti-HBc-positive status as an OBI phase in the natural history of HBV infection.

**Table 4:** HBsAg(-)/anti-HBc(+) liver donor transmitting HBV.

First author	Year	Country or region	Design	Cumulative cases	Followup	Incidence of CHB
Prieto, M (Prieto et al., 2001)	2001	Spain	Retrospective	25	3months	60%
Uemoto, S (Uemoto et al., 1998)	1998	Japan	Retrospective	16	5years	93.75%

In summary, the detection rate of “commonly used” serological methods for OBI diagnosis using highly sensitive HBV DNA tests is mostly below 20%, while the “gold standard” of liver histology has a higher detection rate up to 50% or more. However, liver histology is an invasive examination and is currently not standardized. This indicates that the “common” methods are unreliable for the diagnosis of OBI, while the reliable “gold standard” method is not practical. The mechanism of difficulty in cccDNA clearance, as well as the results of research on the formation, diagnosis, and transmission of HBV through transplantation or transfusion strongly suggest that the possibility of OBI in anti-HBc positive individuals can reach over 90%. Therefore, the “alternative” anti-HBc test is the most reliable and practical marker for OBI diagnosis.

## Author contributions

RX and XW contributed to the initial manuscript writing and literature search. CW, LX, and JX participated in scientific discussions and contributed to the writing of some sections. JX proposed the writing ideas, provided guidance, revised the manuscript, and finalized the paper. All authors contributed to the article and approved the submitted version.
